# The future of public health doctoral education in Africa: transforming higher education institutions to enhance research and practice

**DOI:** 10.1016/S2468-2667(24)00056-2

**Published:** 2024-05-10

**Authors:** Justine Bukenya, Derege Kebede, Henry Mwambi, Muhammed Pate, Philip Adongo, Yemane Berhane, Chelsey R Canavan, Tobias Chirwa, Olufunmilayo I Fawole, David Guwatudde, Elizabeth Jackson, Isabel Madzorera, Mosa Moshabela, Ayoade M J Oduola, Bruno Sunguya, Amadou Sall, Tajudeen Raji, Wafaie Fawzi, ARISE Public Health Education Team

**Affiliations:** a School of Public Health, Makerere University, Kampala, Uganda; b School of Public Health, Addis Ababa University, Addis Ababa, Ethiopia; c School of Mathematics, Statistics, and Computer Science, Durban, South Africa; d Vice Chancellor's Office, University of KwaZulu-Natal, Durban, South Africa; e Harvard T H Chan School of Public Health, Harvard University, Boston, MA, USA; f School of Public Health, University of Ghana, Accra, Ghana; g Association of Schools of Public Health in Africa, Accra, Ghana; h Addis Continental Institute of Public Health, Addis Ababa, Ethiopia; i School of Public Health, Faculty of Health Sciences, University of the Witwatersrand, Johannesburg, South Africa; j Faculty of Public Health, College of Medicine, University of Ibadan, Ibadan, Nigeria; k University of Ibadan Research Foundation, University of Ibadan, Ibadan, Nigeria; l Muhimbili University of Health and Allied Sciences, Dar es Salaam, Tanzania; m Institut Pasteur de Dakar, Dakar, Senegal; n Africa Centers for Disease Control and Prevention, Addis Ababa, Ethiopia

## Abstract

The African Union and the Africa Centers for Disease Control and Prevention issued a Call to Action in 2022 for Africa's New Public Health Order that underscored the need for increased capacity in the public health workforce. Additional domestic and global investments in public health workforce development are central to achieving the aspirations of Agenda 2063 of the African Union, which aims to build and accelerate the implementation of continental frameworks for equitable, people-centred growth and development. Recognising the crucial role of higher education and research, we assessed the capabilities of public health doctoral training in schools and programmes of public health in Africa across three conceptual components: instructional, institutional, and external. Six inter-related and actionable recommendations were derived to advance doctoral training, research, and practice capacity within and between universities. These can be achieved through equitable partnerships between universities, research centres, and national, regional, and global public health institutions.

## Introduction

Over the past 25 years, Africa has made substantial progress on many health indicators, yet continues to face burdens of infectious diseases, malnutrition, child and maternal mortality, non-communicable diseases, and the impacts of climate change.^1^, ^2^ In September, 2022, the Africa Centers for Disease Control and Prevention called to action a New Public Health Order for Africa that included the need to strengthen public health institutions and increase investment in the public health workforce.^3^ This call aligns with Agenda 2063 of the African Union, which provides a framework for sustainable development on the continent.

Public health institutions play a vital part in mitigating national and transnational threats of disease. However, there is a critical shortage of researchers, estimated at 80 researchers per 1 million people in Africa compared with a global average of 1081.^1^ Africa will need at least an estimated 12 500 epidemiologists by 2050 to contribute to global health security and practice.^4^ WHO has laid out a roadmap for building the public health and emergency workforce.^5^ While most African universities offer medical training and Masters of Public Health programmes,^6^ more doctoral and postdoctoral-level training programmes are needed to supply highly trained faculty and research and practice leaders that would further strengthen other graduate programmes and serve as inspiration for those completing undergraduate and master's training.^7^ This is particularly necessary following the COVID-19 pandemic, which underscored the need for more public health professionals in Africa.

Thus, the Africa Research, Implementation Science, and Education (also known as ARISE) Network and partners aimed to assess existing capabilities and opportunities for the enhancement of doctoral-level research and training at public health institutions across the continent. The results were used to develop recommendations for strengthening postgraduate training programmes in Africa.

## Methods and conceptual model

**Figure 1:**
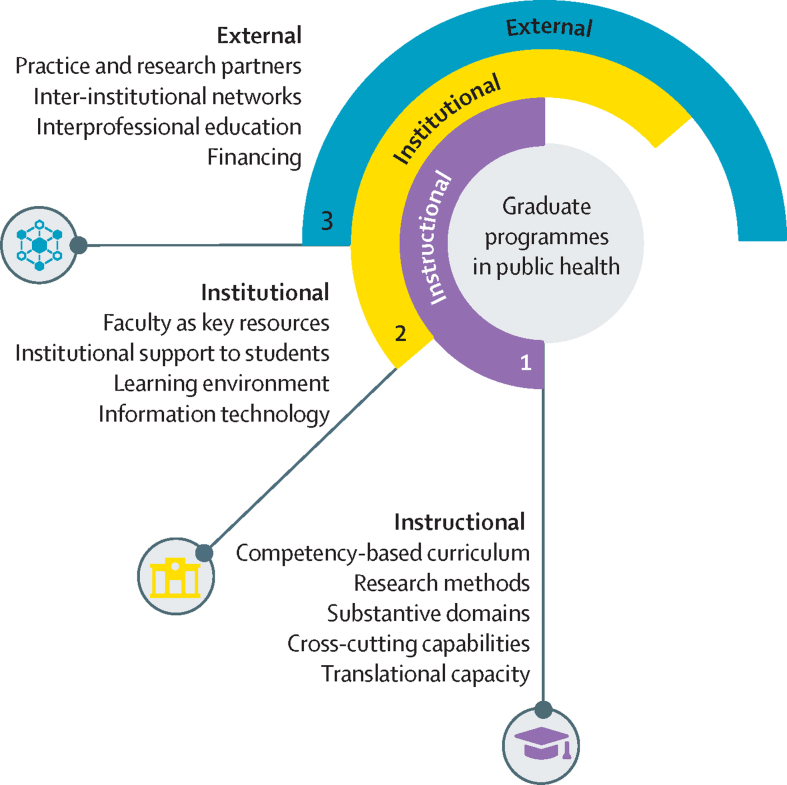
Environmental components of graduate public health programmes

We aimed to describe the existing public health education and doctoral training landscape and identify priority areas for developing innovative and sustainable programmes to advance research and practice in Africa. We developed a conceptual model (figure 1) informed by the 2010 *Lancet* Commission findings including the description of institutional and instructional components of the educational system^8^ and a literature review (appendix pp 5–6). We developed areas of inquiry for a survey based on the three components of our model: instructional, institutional, and external. Questions were adopted from WHO's Health Research System Analysis Initiative.^9^, ^10^

The instructional component focused on existing capacities in learning and knowledge generation, including the existence of and gaps in competency-based curricula for doctoral training. The institutional component assessed the capacity of higher education institutions, particularly elements related to the support of faculty and students and information technology. The external environment component analysed linkages of universities with national research and practice institutions, inter-university networks, interprofessional education, and financing.

The study units of interest were institutions of higher education in all 54 countries across Africa that met the following criteria: a degree-granting institution that has a school or programme of public health or related field and offers a doctoral degree including Doctor of Philosophy (PhD), Doctor of Science (ScD), or Doctor of Public Health (DrPH). Deans and directors of schools or programmes of public health were invited to participate.

The survey was conducted between August, 2021 and October, 2022. Respondents provided informed consent to participate electronically. The survey questionnaire was sent to 119 institutions. The response rate was 61% (73/119), of which 53 institutions in 18 countries reported having doctoral programmes. Finally, 47 institutions with doctoral programmes were included in the analysis after eliminating duplicate and incomplete responses.

48 (91%) of 53 responding programmes offered a PhD or ScD degree. Institutions in Africa have traditionally conferred the PhD degree, structured and targeted at those pursuing predominantly academic and research careers. While the DrPH degree, which focuses on professional public health leadership and practice, is noted as a promising track to advance in Africa,^11^ only 11 (21%) of 53 responding programmes offered a DrPH degree.

## Instructional elements

### Competency-based education

More than half of doctoral programmes (n=27, 59%) in this survey required formal coursework as part of the training, with the other half requiring thesis preparation with elective course offerings. Most programmes (n=34, 74%) noted that core competencies were defined for doctoral training. The adoption of competency-based education, which focuses on what health professionals can do with metrics,^12^ has risen in medicine, nursing, and public health since the publication of the landmark *Lancet* Commission paper.^8^, ^13^

### Methods competence and analytics

**Figure 2:**
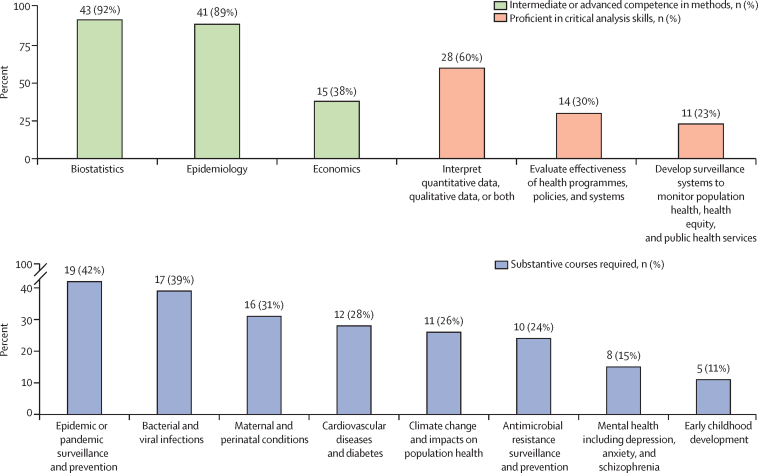
Instructional elements of doctoral training programmes n (%) refers to the number and percentage of programmes surveyed.

In our survey, most programmes required intermediate or advanced competence in biostatistics (n=43, 92%), epidemiology (n=41, 89%), and social and behavioural sciences (n=30, 67%), but less so for economics (n=15, 38%). While most programmes noted graduates would be proficient (n=28, 60%) or at least knowledgeable (n=17, 36%) in the interpretation of quantitative or qualitative data or both (in total n=45, 96%), a smaller proportion deemed their graduates to be similarly competent in other critical analytic skills, including skills to conduct systematic reviews (n=59, 80%) and evaluate the effectiveness of programmes (n=36, 77%; figure 2; appendix pp 8–9).

Analytical skills obtained from essential training in epidemiology and biostatistics are foundational to public health research. Available data call for strengthening competence in the foundations of data science methods to drive discovery, improve outcomes, and reduce costs.

### Substantive domains

In our survey, required course offerings included epidemics and pandemics (n=19, 42%) and maternal and child health (n=16, 31%). Fewer institutions offered courses on emerging and rapidly rising disease conditions including non-communicable diseases, mental health, social determinants of health, or health implications of climate change (figure 2; appendix pp 8–9).

The strong focus on epidemics and pandemics is justifiable given the continued threats from infectious diseases. Competency in basic biomedical sciences is also essential for disease surveillance. While there has been substantial investment in these domains (for example through the H3 Africa Initiative and the Wellcome Trust), additional resources are needed. Given the high burden of non-communicable and climate-related diseases and conditions, greater attention to social and environmental areas is warranted.^14^

### Cross-cutting capabilities

Institutions reported that upon completing their degree, doctoral students were most able to facilitate communication among individuals, groups, and organisations (n=24, 51%; appendix pp 10–11). Other skills were less developed including the ability to create messages to communicate information to influence behaviour (n=17, 32%). Most programmes reported strengths in community engagement (n=43, 94%) and minimal to moderate competency in diversity and inclusion.

Essential aspects of public health training include communication, leadership, diversity and inclusion globally,^15^ financial management, and community engagement. Cross-cutting competencies for public health are crucial, especially in a post-COVID-19 world.^16^, ^17^

### Translational capacities

Two-thirds of programmes expected doctoral candidates to be competent in dissemination and implementation research. 38 (85%) required competencies in definition, rationale, and theory, 36 (79%) in design and analysis, and 34 (76%) in practice-based knowledge and skills (appendix pp 12–13). This includes differentiating between dissemination and implementation research and related areas (n=37, 84%) and assessing and describing the context for effective dissemination and implementation research (n=34, 80%).

Innovations and evidence-based interventions might not be widely implemented to achieve the greatest health impact.^18^, ^19^ Developing expertise in knowledge translation is crucial to increase the uptake of research into policy and action.^20^

## Institutional elements

### Faculty as key resources

34 (64%) respondents reported inadequate salaries and benefits to researchers (appendix pp 14–15), whereas 32 (71%) respondents reported institutions offered sabbaticals to senior faculty and 28 (67%) reported institutions offering mentorship programmes for junior researchers.

The effectiveness of programmes varies depending on the number of faculty; gender balance among faculty and programme leadership; areas of discipline and research focus; financial support for faculty; and access to resources for professional growth. Our findings confirm previous research that limited financial resources, human resources, and national and international collaborations are the main barriers to improving research capacity in Africa.^21^, ^22^ Postdoctoral fellowships provide a pipeline and should be further assessed in future surveys.

### Institutional support to students

**Figure 3:**
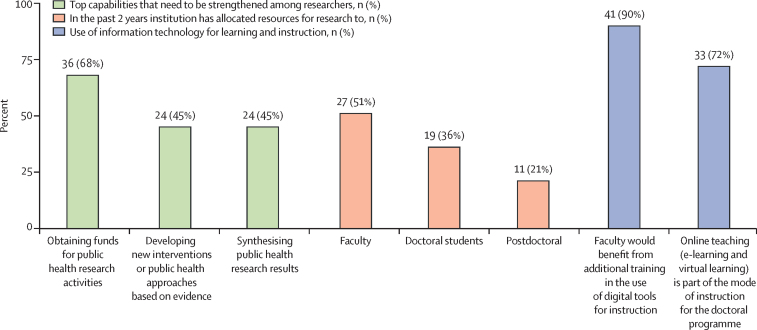
Institutional elements of doctoral training programmes n (%) refers to the number and percentage of programmes surveyed.

In this survey, only 19 (36%) respondents reported institutions allocating resources to doctoral students (figure 3). Primary sources of tuition funding for doctoral students were students themselves (n=24, 51%), the institution (n=12, 26%), and external sources (n=7, 15%). Respondents reported the cost of living was mostly supported by individuals (n=36, 80%). Research support was funded by students (n=19, 41%), the institution (n=10, 22%), and external sources (n=15, 33%).

### Learning environment

Among the top research areas at institutions, communicable, maternal, perinatal, and nutritional conditions were reported by 37 (70%) respondents, and health systems research by 27 (51%). 39 (74%) institutions regarded the national ministry of health among their top three primary intended users of research, whereas 15 (28%) responded sub-national or local health policy or health decision makers.

In our survey results, barriers to research included poorly structured ethical review committees and inadequately trained members. 48 (96%) institutions required ethical review for all research involving human subjects and 41 (82%) had formal ethical review committees (appendix pp 14–15). Notably, almost all the respondents had ethical review committees in place, which is a major improvement from previous surveys.^23^

### Information technology

In our survey, 31 (65%) programmes had computer laboratories. Moreover, 34 (74%) provided access to the latest journals through institutional subscriptions. 40 (85%) institutions had access to broadband internet (appendix pp 14–15).

Online teaching was part of instruction in 33 (72%) programmes. Of these, 22 (69%) introduced online learning since the emergence of COVID-19, and 24 (73%) had dedicated technical personnel. 41 (90%) respondents noted their faculty would benefit from additional training in information technology (figure 3; appendix pp 14–15).

One of the most profound impacts of the COVID-19 pandemic on higher education was a rapid and seemingly permanent shift to online and hybrid learning methods. Online learning encompasses the application of technology in pedagogy and provides the opportunity to increase equity in public health training in Africa.^24^, ^25^, ^26^ African institutions face numerous barriers including access to technology, the cost of reliable high bandwidth internet, information technology human resource capacity, and socio-cultural barriers.^27^ The uptake of hybrid learning can be particularly challenging for institutions in rural areas.^28^

Massive open online courses have been suggested as a promising approach to address quality and equity in higher education.^29^, ^30^, ^31^ Accessibility, applicability, and uptake of open online courses in Africa are mixed.^32^, ^33^ Fees for these courses pose a barrier to access. An information technology strategy and governance structure that prioritises equity and quality and aligns with institutional missions and goals can help drive meaningful investments.^34^, ^35^

### Accreditation

In our survey, 31 (61%) institutions were accredited by a national body. An overview of accreditation of programmes in 39 countries in Africa found that 31 (79%) of the countries surveyed had a health training accreditation programme. There is a need for strengthened regulatory bodies to conduct and enforce public health accreditation measures.^36^ Capacity for the regulation of programmes exists. Several African countries have specialised entities for accreditation of health programmes through the ministry of education or ministry of health. An example is the Field Epidemiology Training Program accreditation process.^37^

## External environment elements

### Networks and partnerships

**Figure 4:**
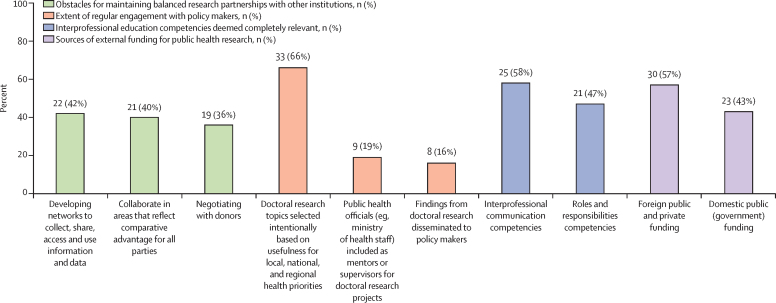
External elements of doctoral training programmes n (%) refers to the number and percentage of programmes surveyed.

In this survey, most institutions were members of a formal network or had partnerships with other research or training institutions. We found variable rates of collaboration with partners within Africa (median 14% of current projects done in collaboration with partners from their region and 10% with partners from other regions of Africa), within Europe (median 17%), and North America (median 19%), with less conducted in collaboration with partners in Asia (appendix pp 16–17). Most institutions reported one or more obstacles to networking, including collaboration in areas that reflect the comparative advantage of all parties (n=21, 40%), sharing benefits equitably (n=19, 36%), and deciding on the use of funds (n=17, 32%). Only 9 (19%) institutions often included public health officials outside the institution as mentors or supervisors, while 8 (16%) regularly disseminated their findings (figure 4).

**Figure 5:**
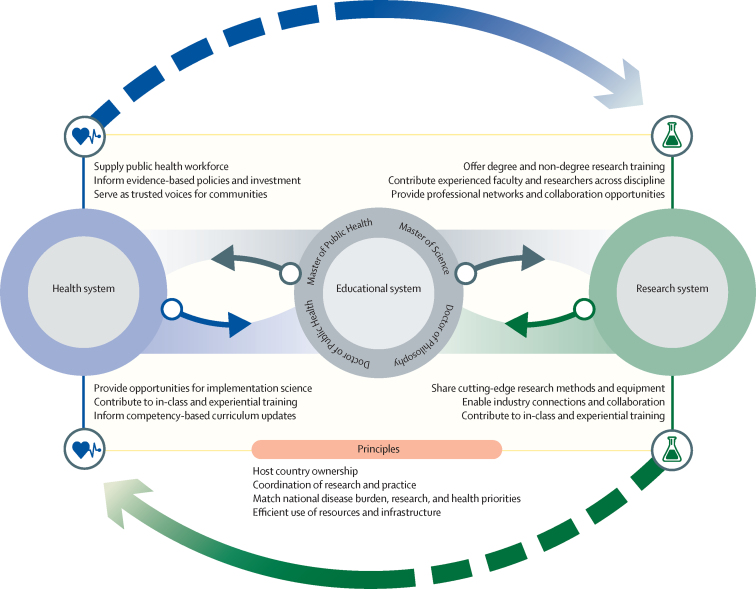
Educational ecosystem for public health research and practice

Figure 5 depicts the key public health degree programmes and the ecosystem of health and research systems. Networks between schools of public health can enhance doctoral training through course offerings, co-mentoring and supervision of students, experiential learning, and collaborative research projects. Although our survey supports high rates of collaboration internationally as has been previously reported,^38^ we also found strong collaboration between African institutions. Several examples of academic and research networks and partnerships exist in Africa (eg, the Africa Centers of Excellence project and the European and Developing Countries Clinical Trials Partnership).

Additionally, National Public Health Institutes are at the forefront of public health preparedness and response in African countries. Partnerships with National Public Health Institutes help ensure that public health research is aligned with national priorities. Many National Public Health Institutes partner with public health schools to offer Field Epidemiology Training Programs that train students to respond to public health needs.^39^, ^40^, ^41^ Partnerships with National Public Health Institutes also facilitate effective translation of research into policy and practice.

Networks could also harness the strengths of the African diaspora, enabling the sharing of comparative insights and strengthening training and research on the continent.^42^, ^43^ While engaging academic partners in high-income countries can be mutually beneficial, it is important to be cognizant of historical imbalances and guard against potential negative effects that have been highlighted in the decolonise global health movement.^44^, ^45^ Voller and colleagues summarised guidelines for North–South research partnerships to address imbalances and structural inequities. They found that partners in the South remain under-represented in guideline development and recommend exploring how guidelines are used in practice.^46^ Implementation of these guidelines could also help address brain drain challenges.

### Interprofessional education

We assessed interprofessional education relevance and incorporation of the five WHO competencies^47^, ^48^ and found that most institutions reported interprofessional competencies as quite or completely relevant, including teams and teamwork (n=39, 84%), interprofessional communication (n=35, 81%), values and ethics for interprofessional practice (n=35, 79%), and roles and responsibilities (n=35, 78%; figure 4; appendix pp 16–17). We found that when interprofessional education was incorporated into training programmes it was mostly informal (n=32, 68%). The top reported barrier to interprofessional education was financial limitations (n=35, 66%).

Our findings are aligned with previous reports indicating adequate curriculum space, funding, and administrative support as key challenges for interprofessional education.^48^, ^49^, ^50^ To successfully implement interprofessional education, there must be cooperation and participation from faculty and students.^49^ Previous studies have found varying levels of enthusiasm and sentiments for interprofessional education training among students and faculty.^51^, ^52^

Systems thinking that enables learning across sectors and the development of graduates who are competent to work in interdisciplinary teams is crucial.^14^ Examples of interprofessional education include One Health and Field Epidemiology Training Programs.^53^

### Financing for public health research and training

Lack of financial resources and funding for research was the most substantial barrier to institutions' ability to perform or sustain public health research activities (n=49, 93%; appendix pp 14–15). The top competency that needs to be strengthened was the ability to obtain funds for research (n=36, 68%; figure 3). Most (n=49, 79%) respondents reported receiving funds for public health research from external sources. The main source was foreign public and private funding (n=30, 57%), followed by domestic public funding (n=23, 43%; figure 4; appendix pp 16–17). Foreign donors included multilateral funds (n=21, 40%), bilateral funds (n=18, 34%), private not-for-profit organisations and foundations (n=18, 34%), and direct funding from foreign universities (n=15, 28%). Public domestic funding was primarily from ministries of health (n=11, 21%) and ministries of education (n=9, 17%).

In 2006, African Union member countries committed to spending at least 1% of gross domestic product on research and development. However, most countries have not achieved this level of funding. Sub-Saharan Africa spent 0·32% of gross domestic product on research and development in 2020.^54^ The International Covenant on Economic, Social, and Cultural Rights states that everyone has the right to benefit from scientific progress, and as noted in General Comment 25, states are therefore obligated to invest in research and education.^55^ Inadequate funding can unintentionally lead to imbalanced relationships with for-profit organisations and other funders whose interests could be potentially in conflict with that of universities.^56^

There is a promising shift in external funding (eg, from the National Institutes of Health, the Wellcome Trust, Grand Challenges, and the Bill & Melinda Gates Foundation) to a model in which programmes are developed and supported within Africa. However, global funders tend to limit overhead or indirect costs provided to institutions in low-income settings, which hinders capacity strengthening. In the USA, negotiated indirect costs have been essential for strengthening the capacity of universities.^57^ Equitable funding mechanisms for public health research and training are foundational for the enhancement of doctoral programmes.

### Summary of findings

In summary, in our survey, we found relatively high inclusion and expected proficiency in the foundational skills of epidemiology and biostatistics, and mixed results in critical analysis skills. Limited attention was given to the rising tide of health priorities related to non-communicable diseases and climate change. Programmes were relatively strong in community engagement and communication skills. There was low proficiency in diversity concepts and health behaviour change communication. Most programmes engage in some form of online learning; the top barrier was a lack of funds and resources. The most common source of funding was foreign and private funding. About half of the programmes considered interprofessional education competencies as completely relevant and a scarcity of collaboration with other researchers and organisations was reported as a top barrier. Most institutions reported high degrees of relevance of research for policy makers. Almost all institutions were members of a formal network or had partnerships with other research or training institutions locally or internationally. On the basis of these trends, we developed a set of recommendations to improve public health training in Africa.

## Recommendations

Six overarching recommendations have emerged towards contributing to transforming higher education institutions to advance public health training, research, and practice. They can be viewed through the instructional, institutional, and external domains of our framework.

### Introduce and expand competency-based education and standard accreditation

Public health programmes should be grounded in core competencies to produce leaders capable of preventing and addressing future health challenges. Future public health leaders must be able to synthesise information from multiple methodologic, substantive, cross-cutting, and translational domains and show effective leadership with interdisciplinary teams. An area for growth and opportunity in advancing public health practice is to increase DrPH programmes, while further strengthening PhD training.

After establishing a competency-based curriculum through university and country-led processes, regular assessment and standard accreditation of these programmes are necessary for quality assurance. Suggested actors include ministries of education (to lead and coordinate), and stakeholders such as ministries of health, the Association of Schools of Public Health in Africa, and universities.

### Accelerate information technology infrastructure transformation at universities

University technology infrastructure needs to be strengthened to increase accessible, well equipped, modern computer laboratories, data storage, and processing capabilities. To create and provide access to effective and efficient digital learning, institutions must make substantial investments in digital systems and digital content. Noting the difficulties that many institutions face in their capacity to do this, institutions have been, and can be, proactive in finding innovative solutions and partnering with industry and local, regional, and global partners.

Strengthening the information technology workforce and additional training for existing faculty and staff are required, and should include addressing computer and internet access for students, especially those in rural areas. Online and hybrid learning can help to equitably increase access to training programmes and can improve the quality of programmes (including innovations in new learning, flipped classrooms, connecting with network partners, and digital infrastructure) and enhance the pedagogical approaches to doctoral teaching. Suggested actors include university leadership with information technology units producing guidelines and policy and governments as key stakeholders.

### Increase financing for capacity building

Increased investment in research and training infrastructure from domestic and global financing is necessary to strengthen African higher education and research institutions. This can be through national, bilateral, regional, and global networks. Financing should be guided by the priorities of each country or region and must be structured to ensure no conflicts of interest exist in financing mechanisms and that funding does not create or reinforce harmful power dynamics. Conflicts of interest are of particular concern for private sector funding, but must be considered regardless of funding sources. A reassessment of the overhead rates allowed by donors is needed to provide for a sustained flow of resources to strengthen the institutional research and training infrastructure. Domestic public financing, private funding from industry, not-for-profit entities, and philanthropy present untapped resources.

To improve research capacity and work towards greater equity in public health research and training, public health programmes must be able to provide sustainable financial support for faculty and doctoral students, formal research training, access to research and publication resources, opportunities for sabbaticals and mentorship programmes, and engagement with international training programmes. Funding for faculty and their research is imperative to retain the workforce needed to train the next generation of public health researchers. Additionally, funding for postdoctoral training opportunities could help incentivise research careers within Africa for graduates. Suggested actors include funding stakeholders at national, regional, and global levels.

### Promote interprofessional education

Interprofessional education is necessary to address pressing public health issues including pandemic preparedness and climate change. Institutions interested in incorporating interprofessional education more formally should consider opportunities to support faculty development in interprofessional education and to build interprofessional education into existing curricula. Also, business and policy schools within universities can play an important part in public health education through partnerships that strengthen training in leadership, strategy, and entrepreneurship to address public health challenges. Enabling cross-registration for courses across schools should be highly encouraged. Developing mapping at a university level to depict where and how one can get credits from core and elective courses would be beneficial. Suggested actors include university leadership and academic programme heads.

### Strengthen links between academic, practice, and research partners

Academic public health networks can promote local and regional exchanges for cross-learning and knowledge transfer. Networks can have a role in reaching consensus on shared degree programme competencies. We recommend universities within a country or region come together to harness their collective strengths. This includes intentional mentorship pairings between different generations of universities and training programmes between academic institutions. Public health associations exist in many countries and can be a resource for network creation and facilitation. Networks would be strengthened by engaging and leveraging national science academies, united through the Network of African Science Academies.

Engaging with National Public Health Institutes and research centres sets the stage for public health research to be aligned with national priorities. Links between public health programmes and regional research centres provide valuable training experiences and enhanced capacity. Regional health institutions such as the Africa Centers for Disease Control and Prevention can provide opportunities for public health students to gain locally contextualised experience in areas of importance. Suggested actors include university leadership, academic programme heads, leadership of public health institutions, the Association of Schools of Public Health in Africa, National Public Health Institutes, and other practice and research centres.

### Implement ongoing tracking and monitoring and sharing of best practices and lessons learned

Higher education institutions would benefit from ongoing evaluation to facilitate continued improvement around advancing public health research and practice. Regular convening of university leaders via communities of practice would facilitate information sharing. This should be done in collaboration with existing groups (eg, Africa Centers for Disease Control and Prevention and the African Union) and leveraging the infrastructure of existing consortia such as the Association of Schools of Public Health in Africa. Engaging with the Association of Schools and Programs of Public Health and with high-income country academic institutions in the spirit of partnership will deepen global interdependence. Strengthening knowledge dissemination through local and regional conferences, symposia, and social media could make the evidence generated within the region benefit the countries and context.

Harmonising doctoral programmes between institutions would be desirable. This could include the alignment of competencies, admission requirements, credit definitions, and academic calendars to facilitate participation in elements of programmes beyond one's institution. This would further allow acceptance of accredited programmes and degrees and facilitate the flow of the workforce between countries in ways that enable a rapid emergency response and contribute to strengthened health and research systems across the continent.^58^ Suggested actors for this recommendation include the Association of Schools of Public Health in Africa leadership and leaders of other relevant regional consortia and global academic partners.

## Conclusion

Higher education and research in public health are vitally important for addressing the burden of disease in Africa. This Health Policy report sought to measure and describe the existing public health training landscape at the doctoral level in Africa and identify priority areas for developing innovative and sustainable programmes. A conceptual framework with three components—instructional, institutional, and external—helped to provide a deep understanding of the training landscape. We provide six recommendations that are intended as a starting point for actionable steps in advancing public health doctoral training to be taken collaboratively towards improved health in Africa. Substantial investment and commitment through equitable partnerships will be required to truly transform higher education institutions towards advancing public health research and practice in Africa.

## Declaration of interests

We declare no competing interests.
