# Supplementary material for: The future of public health doctoral education in Africa: transforming higher education institutions to enhance research and practice
Source: Lancet Public Health. 2024 May 10;9(7):e523–32. doi: 10.1016/S2468-2667(24)00056-2 (PMC11209668; doi:10.1016/S2468-2667(24)00056-2)
Supplement: Supplementary appendix [file mmc1.pdf]

THE LANCET
Public Health

Supplementary appendix

This appendix formed part of the original submission and has been peer reviewed. We post it as supplied by the authors.

Supplement to: Bukenya J, Kebede D, Mwambi H, et al. The future of public health doctoral education in Africa: transforming higher education institutions to enhance research and practice. *Lancet Public Health* 2024; published online May 9. https://doi.org/10.1016/S2468-2667(24)00056-2.

Appendix

The Future of Public Health Doctoral Education in Africa: Transforming Higher Education Institutions to Enhance Research and Practice

Justine Bukenya, MD[1], Derege Kebede*, MD[2], Henry Mwambi*, PhD[3], Muhammed Pate*, MD[4], Philip Adongo*, PhD[5,6], Yemane Berhane*, PhD[7], Chelsey R Canavan, MSPH[4], Tobias Chirwa, PhD[8], Olufunmilayo I. Fawole*, PhD[9], David Guwatudde*, PhD[1], Elizabeth Jackson, MPH[4], Isabel Madzorera, ScD[4], Mosa Moshabela*, PhD[10], Ayoade MJ Oduola*, PhD[11], Bruno Sunguya, PhD[12], Amadou Sall, PhD[13], Tajudeen Raji[14], MD, Wafaie Fawzi*, MBBS, DrPH[4], and the ARISE Public Health Education Team (listed in the appendix)

Table of Contents

ARISE Public Health Education Team	3
Survey Design and Literature Review	5
Supplementary Tables	7

ARISE Public Health Education Team

Adebowale, Ayo S, PhD, University of Ibadan, Nigeria
Aja, Godwin N, DrPH, Adventist International Institute of Advanced Studies, Philippines & Babcock University, Nigeria

Ajuwon, Ademola, PhD, University of Ibadan, Nigeria

Amde, Woldekidan Kifle, PhD, University of the Western Cape, South Africa

Andriamanarivo, Mamy Lalatiana, École doctorale Science de la vie et de la santé, Madagascar

Arung, Willy, PhD, Université de Lubumbashi, Democratic Republic of the Congo

Aseh, Promise Munteh, PhD, Catholic University of Cameroon, Bamenda, Cameroon

Assob, Jules CN, Université de Douala, Cameroon
Ateudjieu, Jerome, Faculté de Medecine et des Sciences Pharmaceutiques, Université de Dschang, Cameroon

Azage, Muluken, Bahir Dar University, Ethiopia

Bajunirwe, Francis, Mbarara University of Science and Technology, Uganda

Belachew, Tefera, MD, Jimma University, Ethiopia

Belouali, Radouane, MD, Mohammed VI University of Sciences and Health, Morocco

Chiabi, Andreas, MD, University of Bamenda, Cameroon

Doumbia, Seydou, MD, University of Sciences, Techniques and Technology of Bamako, Mali

El-Sahn, Amel AbdelFattah , DrPH, Alexandria University, Egypt

Eldoom, Ekram Adam, PhD, Alzaeim Alazhari University, Sudan

English, Rene, PhD, Stellenbosch University, South Africa

Faye, Adama, MD, Université Cheikh Anta Diop de Dakar, Sénégal

Frumence, Gasto, PhD, Muhimbili University of Health and Allied Sciences, Tanzania

Gelaye, Kassahun Alemu, PhD, University of Gondar, Ethiopia

Humuza, James, PhD, University of Rwanda, Rwanda

Igumbor, Jude, PhD, University of Witwatersrand, South Africa

Inah, Simon Alain, MPH, University of Calabar, Nigeria

Kaaya, Ephata, PhD, Kilimanjaro Christian Medical University College, Tanzania

Kamgno, Joseph, MD, Faculté de Médecine et des Sciences Biomédicales, Cameroon

Katangolo-Nakashwa, Ndasilohenda, PhD, University of Namibia , Namibia

Khalis, Mohamed, Mohammed VI University of Health Sciences, Morocco

Kibusi, Stephen, PhD, University of Dodoma, Tanzania

Kruger, Willem, University of the Free State, South Africa
Kulimba, Desire Mashinda, Ecole de Sante Publique de Kinshasa, Democratic Republic of the Congo

Likeng, Julienne Louise Ngo, Catholic University of Central Africa, Cameroon

Mariam, Damen Haile, MD, Addis Ababa University, Ethiopia

Matsungo, Tonderayi M, PhD, University of Zimbabwe, Zimbabwe

Michelo, Charles, PhD, Nkwazi Research University, Zambia

Mitonga, Honore Kabwebwe, PhD, University of Namibia, Namibia

Mokwena, Kebogile, MSc, Sefako Makgatho Health Sciences University, South Africa

Motbainor, Achenef, PhD, Bahir Dar University, Ethiopia

Moustafa, Noha Shawky Aly, DrPH, Alexandria University, Egypt

Musonda, Patrick, University of Zambia, Zambia

Mustafa, Nazik Eltayeb Musa, PhD, University of Khartoum, Sudan

Muula, Adamson S, PhD, Kamuzu University of Health Sciences, Malawi

Muyer, Marie-Claire Muel Telo, Ecole de santé publique de Kinshasa, Democratic Republic of the Congo

Myer, Landon, MD, University of Cape Town, South Africa

Naidoo, Saloshni, PhD, University of KwaZulu-Natal, South Africa

Nani, Samira, Hassan II University, Casablanca, Morocco

Navti, Lifoter Kenneth, PhD, Catholic University of Cameroon, Bamenda, Cameroon

Negeri, Keneni Gutema, Hawassa University, Ethiopia

Nejjari, Chakib, Euromed Research Center, Euromed University of Fes, Fes, Morocco

Nkoum, Benjamin Alexandre, Université Catholique d'Afrique Centrale, Cameroon

Ntakirutimana, Theoneste, PhD, University of Rwanda, Rwanda

Obtel, Majdouline, MD,University of Mohammed V in Rabat, Morocco

Ogaji, Daprim, University of Port Harcourt, Nigeria

Oljira, Lemessa, PhD, Haramaya University, Ethiopia

Roberto, Ana Sofia, BA, Eduardo Mondlane University, Mozambique

Salawu, Mobolaji M, MPH, University of Ibadan, Nigeria

Sangho, Hamadoun, MD, University of Sciences, Techniques and Technology of Bamako, Mali

Schneider, Helen, PhD, University of the Western Cape, South Africa

Sevene, Esperança, MD, Eduardo Mondlane University, Mozambique

Sherfi, Huda A, PhD, Ahfad University for Women, Sudan

Zyambo, Cosmas, MD, The University of Zambia, Zambia

Tahir, Hanan, PhD, The University of Medical Sciences and Technology, Sudan

Torpey, Kwasi, PhD, University of Ghana, Ghana

Voce, Anna S, PhD, University of KwaZulu-Natal, South Africa

Vuai, Said A.H., PhD, University of Dodoma, Tanzania

Yenesew, Muluken Azage, PhD, Bahir Dar University, Ethiopia

Youlyouz-Marfak, Ibtissam, Université Hassan 1er de Settat. Maroc, Morocco

Survey Design and Literature Review

ARISE was established in 2014 by leading African institutions, the Africa Academy for Public Health, and Harvard T.H. Chan School of Public Health, and seeks to advance public health research and education through equitable South-South collaboration and triangular South-South-North cooperation. The research team comprising partners at the ARISE Network, Association of Schools of Public Health in Africa (ASPHA), and the Africa CDC developed the survey tool, compiled the list of institutions that were invited to participate, and led the analyses and development of the paper, in collaboration with all respondents of the survey. Furthermore, we carried out a literature review to complement the findings from our survey

Survey: The survey was based on three components that impact graduate public health programs as denoted in the Conceptual Framework: instructional, institutional, and external. The instructional component focused on existing capacities in learning and knowledge generation. Data collection areas included the existence and gaps in competency-based curricula for doctoral training, including methodological and substantive areas. The survey covered levels of training provided in methodological areas such as biostatistics, epidemiology, informatics, and data science, as well as substantive areas such as infectious diseases, non-communicable diseases, and nutrition. We also collected information on translational capacities to link research with policy and practice, including how implementation science and translational skills are developed and deployed within doctoral programs. Finally, data collection on instructional components also included crosscutting capabilities questions such as communication skills; diversity, equity, and inclusion; partnerships, and finance skills, as well as the diversity and inclusion competency expected from graduates using a comprehensive list of D&I competencies.

The institutional component of the survey assessed the role of higher education institutions in supporting and promoting public health doctoral training programs. Data collection areas included the existence of a dedicated office and/or personnel for research support and development; the presence of an institutional review board for ethical review and approval of research studies; the level of ongoing high-quality research that is available to doctoral trainees for applied dissertation work; the existence and utilization of digital support systems, such as computing and data storage; and the utilization of information technology in advancing doctoral training programs, such as online or blended learning approaches.

The external environment component of the survey describes and analyzes financing for education and research. This included the availability of funds for early-stage research projects and to support cross-discipline work, as well as the availability of funds for tuition and living expenses for students and postdoctoral researchers. Data collection also included inter-professional education such as the opportunities for dual degree programs in medicine or nursing and public health. This component also included the role of governance and leadership in the development and success of doctoral programs, including the commitment to and implementation of gender equity and diversity strategies at institutional levels; and participation in coalitions, collaborations, and networks.

The list of institutions included members of the ARISE Network, the Association of Schools of Public Health in Africa (ASPHA), the WHO Health Research System Analysis (HRSA) initiative, and others identified through an internet search. The survey was pilot-tested at two institutions and further refined. An invitation to participate in the study was emailed in English and French. Reminder emails were sent to non-respondents every 3-6 weeks through personal network contacts.Data obtained from 47 institutions with doctoral programs were included in the analysis after accounting for duplicate or incomplete responses (Appendix Table 1).

Literature Review: To further inform the current effort, we conducted a review of available literature on the existing state of doctoral public health training in Africa over the past 20-year period from 2000-2020 (see Box 1 for search terms). The search was conducted in October 2020 using the PubMed database and returned 168 citations. A title review resulted in 74 relevant citations that underwent abstracts' review. Following the review of abstracts, 17 articles were retrieved. We further reviewed the reference lists from these articles to capture the most relevant information to inform our study.

Box 1: Search strategy and selection criteria:

Search terms:

(("Africa"[Mesh])OR("Africa"[Title/Abstract]))AND(("doctoral"[Title/Abstract])OR("graduate"[Title/Abstract])OR("PhD"[Title/Abstract])OR("Ph.D"[Title/Abstract]))AND(("education"[Title/Abstract])OR("training"[Title/Abstract])OR("Education,Graduate"[Mesh])OR("Education,PublicHealth Professional"[Mesh]))AND(("PublicHealth"[Mesh])OR("PublicHealth"[Title/Abstract])OR("Students,PublicHealth"[Mesh])OR("Schools,PublicHealth"[Mesh]))AND (2000:2020[pdat])

Language used: English

Date ranges used: 2000-2020

Supplementary Tables

Appendix Table 1: Institutions with Public Health Doctoral Programs per Country (based on survey respondents)

	Number of Programs
Cameroon	5
Côte d'Ivoire	1
Democratic Republic of the Congo	2
Egypt	1
Ethiopia	6
Ghana	1
Madagascar	1
Malawi	1
Mali	1
Morocco	5
Mozambique	1
Namibia	1
Nigeria	4
Rwanda	1
Senegal	1
South Africa	7
Sudan	4
Uganda	2
United Republic of Tanzania	4
Zambia	3
Zimbabwe	1

Appendix Table 2. Instructional Competencies in Methods, Analytics, and Substantive Areas

Competence in methods	Intermediate/ Advanced	Introductory	None
Biostatistics	43 (92)	1 (2)	3 (6)
Epidemiology	41 (89)	3 (7)	2 (4)
Economics	15 (38)	9 (23)	16 (40)
Social and Behavioral Science	30 (67)	10 (22)	5 (11)
Competence in critical analysis skills	Proficient N (%)	Knowledgeable N (%)	Limited N (%)
Analyze the comparability of data	33 (72)	12 (26)	1 (2)
Interpret quantitative and/or qualitative data	28 (60)	17 (36)	2 (4)
Retrieve evidence from multiple sources	23 (49)	18 (38)	6 (13)
Conduct a systematic literature review	18 (39)	19 (41)	9 (20)
Evaluate effectiveness of health programs, policies, and systems	14 (30)	22 (47)	11 (23)
Develop evidence-based strategies for improving health outcomes	12 (26)	24 (51)	11 (23)
Develop surveillance systems to monitor population health, health equity, and public health services.	11 (23)	19 (40)	17 (36)
Substantive courses offered	Required N (%)	Elective N (%)	Not offered N (%)
Data science and informatics	25 (53)	16 (34)	6 (13)
Health services delivery research	23 (51)	5 (11)	17 (38)
Epidemics/pandemics surveillance and prevention	19 (42)	9 (20)	17 (38)
Health policy and systems research	19 (42)	10 (19)	16 (37)
Bacterial and viral infections	17 (39)	8 (18)	19 (43)
Nutritional deficiencies	15 (34)	9 (21)	20 (46)
Lifestyle factors including smoking, physical activity, diet, sexual practices	14 (32)	9 (21)	21 (48)
Maternal and perinatal conditions	16 (31)	9 (20)	21 (46)
Social determinants of health including poverty, education, gender	13 (29)	10 (22)	22 (49)
Cardiovascular diseases and diabetes	12 (28)	7 (13)	24 (56)
Climate change and impacts on population health	11 (26)	6 (14)	25 (60)
Antimicrobial resistance surveillance/prevention	10 (24)	8 (19)	24 (51)

Injuries, intentional and unintentional	10 (19)	9 (17)	24 (56)
Mental health including depression, anxiety, schizophrenia	8 (15)	9 (17)	28 (62)
Early childhood development	5 (11)	5 (11)	35 (78)
Malignant neoplasms and cancer	6 (11)	9 (17)	30 (67)
Agricultural practices and food safety	4 (10)	5 (13)	31 (78)
Other neuro-psychiatric conditions	4 (10)	10 (19)	32 (70)

Appendix Table 3. Instructional Competencies in Cross-cutting Skills

Graduates are able to	Completely N (%)	Moderately N (%)	Minimal N (%)
Communicate via written	38 (81)	6 (13)	3 (6)
Communicate via oral	33 (70)	10 (21)	4 (9)
Communicate via visual	20 (43)	17 (31)	10 (21)
Communicate via social media	14 (30)	12 (29)	19 (40)
Apply ethical principles in accessing, collecting, analyzing, using, maintaining, and disseminating data and information	40 (89)	4 (9)	1 (2)
Make evidence-based decisions for research agenda and implementation	31 (69)	12 (27)	2 (4)
Advocate with policymakers for the use of evidence in decision making that affects the health of a community	25 (57)	16 (36)	3 (7)
Describe roles of public health, health care, and other partners in improving community health	25 (53)	14 (30)	8 (17)
Facilitate communication among individuals, groups, and organizations	24 (51)	16 (35)	4 (9)
Select approaches for disseminating public health data and information (e.g. social media, newspaper, newsletters, journals, town hall meetings, libraries, neighborhood)	17 (36)	19 (40)	11 (23)
Guide an organization in setting communication goals, objectives and priorities, including risk communication during epidemics/pandemics	17 (36)	15 (32)	15 (32)
Create messages to influence behavior and improve health (e.g. use social marketing methods, consider behavioral theories)	17 (32)	15 (32)	15 (32)
Address diversity of individuals and populations when implementing policies, programs, and services	15 (32)	14 (30)	18 (38)
Recognize contribution of diverse perspectives in developing, implementing and evaluating policy, programs, services	13 (28)	19 (40)	15 (32)
Describe ways diversity may influence policies, programs, services and health of a community	7 (15)	23 (49)	17 (36)
Graduates are expected to be able to:	Yes N (%)		

Collaborate with community partners to improve health in a community	43 (94)
Engage community members to improve health in a community	43 (93)
Students conduct community-based participatory research	29 (62)
Students have opportunities to manage project budgets during training	25 (61)
Program requires instruction in finance and budgeting	15 (28)

Appendix Table 4. Competencies on Dissemination and Implementation

	N	(%)
Definition, Rationale, Theory, and Approaches (Mean)		(85)
Identify core elements of effective interventions	43	(96)
Identify appropriate conceptual models, frameworks, or program logic for dissemination and implementation change	43	(94)
Formulate methods to address barriers of dissemination and implementation of research	34	(81)
Identify the potential impact of disseminating, implementing, and sustaining effective interventions	39	(85)
Differentiate between dissemination and implementation research and other related areas, such as efficacy	37	(84)
Describe a range of dissemination and implementation strategies, models, and frameworks	36	(80)
Describe skills needed to conduct dissemination and implementation research (e.g., mixed-method experience, economic, organizational policy, clinical	36	(80)
Assess and describe the context for the effective dissemination and implementation research (setting characteristics, culture, capacity, and readiness)	34	(80)
Competencies on Design & Analysis (Mean)		(79)
Describe the application and integration of mixed-method (quantitative and qualitative) approaches in dissemination and implementation research	43	(92)
Identify and measure outcomes that matter to stakeholders, adopters, and implementers	39	(89)
Describe the core components of external validity and their relevance to the dissemination and implementation of research	39	(87)
Identify common dissemination and implementation measures and analytic strategies relevant for research questions	38	(84)
Identify and articulate the trade-offs between different study designs for dissemination and implementation of research	37	(84)
Identify and articulate the interplay between policy and organizational processes in dissemination and implementation	35	(81)
Effectively integrate the concepts of sustainability and the rationale behind them in dissemination and implementation study design	32	(75)
Incorporate methods of economic evaluation (e.g. implementation costs, cost-effectiveness) in study design	28	(65)
Evaluate and refine innovative scale-up and spread methods (e.g., technical assistance, interactive systems, novel incentives, and "pull" strategies)	23	(53)
Practice-Based Competencies (Mean)		(76)
Describe the importance of incorporating the perspectives of different stakeholder groups (e.g., patient/family, employers,	41	(89)

payers, healthcare settings, public organizations, community, and policymakers)		
Identify a process for adapting an intervention for implementation research	38	(88)
Identify and apply techniques for stakeholder analysis and engagement when implementing evidence-based practices	37	(86)
Identify and develop sustainable partnerships for Dissemination and Implementation of research	39	(85)
Use evidence to evaluate and adapt strategies for specific populations, settings, contexts, resources, and capacities	37	(84)
Describe the concept and measurement of fidelity	35	(83)
Identify sites to participate in Dissemination and Implementation studies, and negotiate or provide incentives to secure their involvement	35	(82)
Describe the appropriate process for eliciting input from community-based practitioners for adapting an intervention	37	(82)
Determine when engagement in participatory research is appropriate	32	(76)
Explain how to maintain the fidelity of original interventions during the adaption process	29	(71)
Articulate the strengths and weaknesses of participatory research	34	(7)

Appendix Table 5. Institutional Support for Public Health Research and Training

Top significant barriers to the institution's ability to perform or sustain public health research activities	N	%
Lack of financial resources/funding for research	49	(93)
Poor or inadequate salary and benefits of health researchers	34	(64)
Limited or lack of collaboration with other researchers, institutions, organizations (e.g. Ministry of Health, hospitals, laboratories, etc)	34	(64)
The institution offers the following to improve research capacity:		
Provides formal research training for employees	34	(76)
Offers sabbaticals	32	(71)
Provides mentorship program for all research staff	28	(67)
Top capabilities that need to be strengthened:		
Obtaining funds for public health research activities	36	(68)
Developing new interventions or public health approaches based on evidence	24	(45)
Synthesizing public health research results (e.g., research syntheses)	24	(45)
Conducting quantitative programming for data analyses (e.g., SAS, SPSS, STATA)	23	(43)
Managing public health research projects or activities	20	(38)
Using appropriate qualitative collection and analysis approaches	19	(36)
Preparing and submitting abstracts and papers for publications and presentations	18	(34)
Preparing materials (e.g., media and policy briefs) for non-scientific audiences	16	(30)
Developing research proposals including research questions, hypotheses, methodology	15	(28)
Conducting stakeholder analysis or political mapping exercises	13	(25)
Reviewing and setting research priorities with communities or other beneficiaries	12	(23)
Training others to conduct public health research (formal or non-formal courses)	10	(19)
Reviewing and monitoring ethical aspects of public health research projects	5	(9)
In the past two years institution has allocated resources for research to:		
Faculty	27	(51)
Doctoral students	19	(36)
Postdocs	11	(21)
Top public health research areas at the institution		
Research on specific areas of communicable, maternal, perinatal and nutritional condition	37	(70)
Research on health systems	27	(51)
Research on exposure, risk factors, or determinants that contribute to health.	25	(47)
Research on specific areas of non-communicable diseases	24	(45)

Research on climate change and health	1	(2)
Research on refugees, natural disasters, and emergencies	2	(4)
Institution's top most important contributions		
Publishing articles in peer-reviewed scientific journals	43	(81)
Conducting operational or evaluative research	25	(47)
Producing new knowledge	24	(45)
Influencing health policies or programs	22	(42)
Use of Information Technology		
Faculty would benefit from additional training in the use of digital tools for instruction	41	(90)
Broadband access is available at the institution	40	(85)
Faculty and lecturers have experience in online learning	38	(81)
Institution provides access to the latest journals through subscription or programs such as HINARI	34	(74)
There is dedicated personnel to assist with technological issues for online learning	24	(73)
Online teaching (e-learning, virtual learning) is part of mode of instruction for the doctoral program	33	(72)
Online learning was introduced after the COVID-19 pandemic	22	(69)
Institution has a computer laboratory that is accessible to doctoral students	31	(65)
Supervisors and faculty often connect remotely with students using digital platforms	19	(40)
Research seminars are offered often with remote access (e.g. webinar)	14	(30)
Computers (desktop or laptop) are provided for doctoral students	13	(25)
Software staff have access to:		
Statistical packages (e.g. STATA, SPSS, SAS)	44	(83)
Spreadsheets (e.g. Excel)	43	(81)
Bibliographic/referencing (e.g. Endnote, Procite, Mendeley, Reference Manager)	36	(68)
Qualitative analysis (e.g. NUD*IST, NVivo, Dedoose, Ethnograph)	22	(42)
Surveying (e.g. SurveyCTO, RedCap, Open Data Kit)	14	(26)
Project management software	4	(8)
Simulation software	4	(8)
Ethical Review Capacity		
Ethical review is required for all research involving human subjects	48	(96)
Institution has a formal ethical review committee	41	(82)
Institution has at least one staff person who is responsible for research ethics	38	(76)
Diversity		
Institutions with an office or at least one staff member focused on diversity	14	(34)

Appendix Table 6. External Elements of Public Health Programs

Institutions with any collaborations within the past two years with partners according to geographic location	N (%)	Median % of research conducted with partners from region	
North America including Canada and USA	28 (53)		19
Europe	30 (57)		17
Own region	39 (74)		14
Other regions of Africa	24 (45)		10
East Asia, including Japan, India, others (excluding China)	14 (26)		3
China	13 (25)		0
Obstacles for maintaining balanced research partnerships with other institutions			
N (%)			
Developing networks to collect, share, access and use information and data	22 (42)		
Collaborate in areas that reflect comparative advantage of all parties	21 (40)		
Negotiating with donors	19 (36)		
Sharing the materials and immaterial benefits equitably (i.e. profits, scientific recognition)	19 (36)		
Deciding on the use of funds	17 (32)		
Creating transparency	14 (26)		
Deciding on the priorities and objectives together	14 (26)		
Negotiating through different perceptions, conflicts and differences (dispute resolution)	14 (26)		
To what extent are	Rarely N (%)	Sometimes N (%)	Often N (%)
Doctoral research topics selected intentionally based on usefulness for local/national/regional health priorities	4 (8)	13 (26)	33 (66)
Findings from doctoral research disseminated to policymakers	7 (14)	34 (64)	8 (16)
Public health officials (e.g., Ministry of Health staff) included as mentors or supervisors for doctoral research projects	7 (15)	32 (67)	9 (19)
Top primary intended audiences/users of public health research	N (%)		
National Ministry of Health	39 (74)		
Sub-national or local health policy or health decision-makers	15 (28)		
Academic or research institutes, university hospitals	34 (64)		
National offices of international organizations	14 (26)		

Hospitals and other health care facilities	19 (36)		
Other national ministries	2 (4)		
Relevance of IPE competencies	Somewhat N (%)	Quite N (%)	Completely (N (%)
Values and ethics for interprofessional practice	9 (21)	11 (25)	24 (54)
Roles/Responsibilities Competencies	10 (22)	14 (31)	21 (47)
Interprofessional Communication Competencies	8 (19)	10 (23)	25 (58)
Teams and Teamwork Competencies	7 (15)	8 (17)	31 (67)
To what extent does the public health doctoral training cover the following components for inter-professional education?	Somewhat N (%)	Consistently N (%)	
Teamwork	16 (38)	26 (62)	
Leadership styles and skills	23 (54)	20 (47)	
Effective communication	16 (37)	27 (63)	
Building rapport	22 (56)	17 (44)	
Managing conflict	26 (65)	14 (35)	
Equity between participating collaborators	18 (47)	20 (38)	
Primary sources of domestic public funding	N	%	
Ministry of Health	11	(21)	
Ministry of Education	9	(17)	
Ministry of Science and Technology	8	(15)	
National research funding agency	9	(17)	
Other Ministries or Government entities	6	(11)	
Private industry, corporations, insurance enterprises	4	(8)	
Private not-for-profit organizations and foundations	7	(13)	
Philanthropy from individual donors	2	(4)	
Primary sources of foreign funding			
Bilateral funds (direct funding from foreign governments)	18	(34)	
Multilateral funds (WHO, United Nations, World Bank, etc.)	21	(40)	
Private not-for-profit organizations and foundations	18	(34)	
Sources of external funding for public health research	N	%	
Foreign public and private funding	30	(57)	
Domestic public (government) funding	23	(43)	
No external funding	11	(21)	
Domestic private sector	7	(13)	
